# *Burkholderia *Hep_Hap autotransporter (BuHA) proteins elicit a strong antibody response during experimental glanders but not human melioidosis

**DOI:** 10.1186/1471-2180-7-19

**Published:** 2007-03-15

**Authors:** Rachaneeporn Tiyawisutsri, Matthew TG Holden, Sarinna Tumapa, Sirirat Rengpipat, Simon R Clarke, Simon J Foster, William C Nierman, Nicholas PJ Day, Sharon J Peacock

**Affiliations:** 1 Faculty of Tropical Medicine, Mahidol University, Bangkok, Thailand; 2 Faculty of Allied Health Sciences, Chulalongkorn University, Bangkok, Thailand; 3 The Wellcome Trust Sanger Institute, Hinxton, Cambridge, CB10 1SA, UK; 4 Faculty of Sciences, Chulalongkorn University, Bangkok, Thailand; 5 Department of Molecular Biology & Biotechnology University of Sheffield, Western Bank, Sheffield, S10 2TN, UK; 6 The Institute for Genomic Research, Rockville, Maryland 20850, USA; 7 The George Washington University School of Medicine, Department of Biochemistry and Molecular Biology, Washington DC 20037, USA; 8 Center for Clinical Vaccinology and Tropical Medicine, Nuffield Department of Clinical Medicine, University of Oxford, Churchill Hospital, Oxford, OX3 9LJ, UK

## Abstract

**Background:**

The bacterial biothreat agents *Burkholderia mallei *and *Burkholderia pseudomallei *are the cause of glanders and melioidosis, respectively. Genomic and epidemiological studies have shown that *B. mallei *is a recently emerged, host restricted clone of *B. pseudomallei*.

**Results:**

Using bacteriophage-mediated immunoscreening we identified genes expressed *in vivo *during experimental equine glanders infection. A family of immunodominant antigens were identified that share protein domain architectures with hemagglutinins and invasins. These have been designated *Burkholderia *Hep_Hag autotransporter (BuHA) proteins. A total of 110/207 positive clones (53%) of a *B. mallei *expression library screened with sera from two infected horses belonged to this family. This contrasted with 6/189 positive clones (3%) of a *B. pseudomallei *expression library screened with serum from 21 patients with culture-proven melioidosis.

**Conclusion:**

Members of the BuHA proteins are found in other Gram-negative bacteria and have been shown to have important roles related to virulence. Compared with other bacterial species, the genomes of both *B. mallei and B. pseudomallei *contain a relative abundance of this family of proteins. The domain structures of these proteins suggest that they function as multimeric surface proteins that modulate interactions of the cell with the host and environment. Their effect on the cellular immune response to *B. mallei *and their potential as diagnostics for glanders requires further study.

## Background

*Burkholderia mallei *is the causative agent of glanders, a serious Gram-negative infection that predominantly affects horses and other equines [1]. Natural *B. mallei *infection has largely been eradicated and human infection is extremely rare, but renewed interest in this organism parallels its classification as a category B biothreat agent. There is a need to develop an effective vaccine for individuals at risk of exposure from deliberate release, and understanding the immune response elicited during infection with *B. mallei *is central to this process. The relative importance of cellular versus humoral responses in the development of protective immunity against *B. mallei *is under investigation. Experimental evidence indicates that both play a role, but that stimulation of a Th1-like immune response may be important for protection [2-5]. One study of murine monoclonal antibodies (mAbs) against *B. mallei *administered to mice prior to a lethal aerosol challenge reported non-sterilizing protection [6]. Passive protection in the mouse model has also been described for the highly related *B. pseudomallei *using murine mAbs specific for *B. pseudomallei *polysaccharide [7].

The development of improved diagnostic tests for the diagnosis of glanders is also a necessity. PCR has been described for the detection of *B. mallei *during acute presentation [8-10], but the antibody response to infection is poorly understood and there are no serological tests currently recommended for the diagnosis of glanders in patients who have been partially or fully treated and in whom *B. mallei *is not detectable. Humoral immune response profiling to infection is a logical approach to the evaluation of immunogenic antigens, and has the ability to define novel targets for both diagnostics and vaccines. Profiling using proteome microarrays has been described for a vaccinia virus proteome consisting of 185 individual viral proteins and used to determine Ab profiles in serum from vaccinia virus-immunized humans, primates, and mice [11]. This technology has not been described in the literature to date for antibody profiling in response to infection with *B. mallei *or *B. pseudomallei*, although polysaccharide microarray technology has been described for the detection of *B. pseudomallei *and *B. mallei *antibodies to capsular antibodies [12]. An alternative strategy for immunoscreening is the use of a bacteriophage expression library. This has recently been used to detect *in vivo *expressed antigens of *Staphylococcus aureus *and to utilize this information to develop a vaccine to protect against experimental nasal carriage [13]. Although complex molecules such as LPS will not be represented, this technique offers an immediately available, rapid and simple means of identifying and testing putative candidate vaccines and targets for serological diagnostic tests. We have applied bacteriophage-mediated immunoscreening to identify antibody responses to *in vivo *expressed genes during experimental equine glanders infection, and have compared this with those elicited during human melioidosis.

## Results

### *B. mallei *expression library screening with glanders serum

The *B. mallei *expression library was individually probed with sera from two experimentally infected horses taken seven days after intra-tracheal bacterial inoculation with *B. mallei *ATCC 23344. This equine glanders model has been described previously [14]. Positive plaques were picked and purified by additional rounds of screening. A total of 207 positive clones were identified and partially sequenced. These corresponded to 71 different loci (comprising a contiguous DNA region from single or overlapping clones) containing 228 known or putative protein coding sequences (CDSs) of *B. mallei*. Additional file 1 provides full details of the *B. mallei *antigens recognized by one or both of the infected horse sera. The distribution of loci was not equal between the two chromosomes; 50 loci were present on chromosome 1, and 21 loci were present on chromosome 2 in *B. mallei *ATCC 23344.

The specificity of these results for experimental glanders infection as opposed to the presence of pre-existing cross reactive antibodies to other bacterial species was tested by screening serum taken from one healthy experimental horse prior to infection with *B. mallei*. A total of 20 screenings failed to reveal any positive plaques (data not shown). This strongly suggests that the antibodies detected in sera from the two infected horses are specific to the experimental *B. mallei *infection.

Four loci were highly over-represented amongst the positive clones; these are shown in Table 1. These four loci together represented 110 (53%) of all positive clones. The most frequent was locus number 14, which was represented in 40 clones, followed by locus 10 (24 clones), locus 54 (24 clones) and locus 61 (22 clones). This compares with the fifth most frequent locus, which occurred only 6 times (Additional file 1). Both horse sera recognized each of the four common loci. The specificity of this result was confirmed by Western blot of one representative clone of each of the four loci (data not shown).

The CDSs in each of the four common loci were examined to identify the putative antigen responsible for immune stimulation. This was achieved by mapping overlapping clones at each locus. A complete or partial copy of BMA0840 was present in all clones of locus 10; the same was true for BMA1027 in locus 14, BMAA0649 for locus 54 and BMAA1324 for locus 61. Furthermore, BMA0840 was the only gene present in 6 independent clones, BMA1027 was the only gene present in 14 clones, BMAA0649 was present alone in 12 clones, and BMAA1324 was present alone in 8 clones. Based on these observations, we propose that these CDSs encode immuno-stimulatory proteins during experimental equine infection. We cannot infer from this that all four genes were expressed during equine infection; the presence of highly homologous regions between the four proteins could lead to antibody cross-reactivity. Furthermore, our results do not exclude the possibility that additional CDSs encoding surface-expressed proteins present in the four commonly represented loci also contributed towards the antibody response. For example, BMA0841 (locus 10) encodes a putative OmpA family protein, and BMAA1323 (locus 61) encodes a putative outer membrane protein OmpA/SmpA/OmlA family.

### *B. mallei *Burkholderia Hep_Hag autotransporter (BuHA) proteins

**Figure 1:**
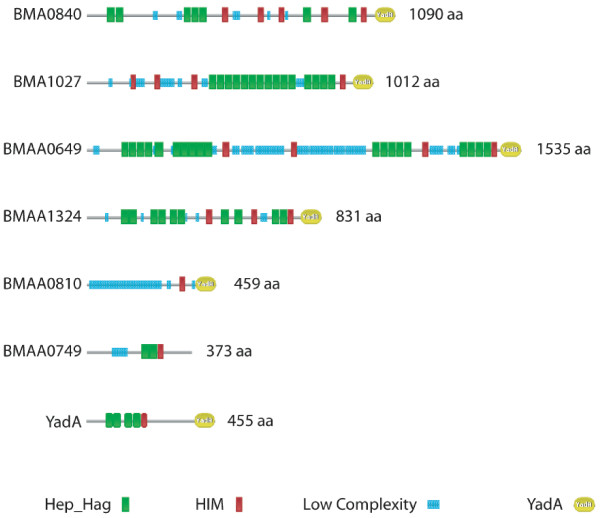
**Domain architecture of *Burkholderia mallei *BuHA proteins**. Pfam [36] domain organization of 4 immunodominant BuHA proteins of *B. mallei*, and 2 additional CDSs from *B. mallei *that contain Hep_Hag domains. Also included is the YadA protein from *Yersinia enterocolitica *(accession number YADA1_YEREN). Keys are shown for the domains in the bead diagrams.

Three of the four putatively immunodominant antigens were annotated as hemagglutinin family proteins, and the fourth was annotated as a putative outer membrane protein (Table 1). The domain organization of these proteins was defined and compared using the Pfam protein family database [15]. All four proteins contain a C-terminal YadA domain (Pfam domain PF03895), together with several HIM (Pfam domain PF05662) and Hep_Hag (Pfam domain PF05658) domains (Figure 1). The Hep_Hag domain is a repetitive region comprising a 7 amino acid repeat that is found in haemagglutinins and invasins. The HIM domain is another short motif (~20 amino acids) that is often found in conjunction with the Hep_Hag motif. The YadA domain is composed of approximately 120 amino acids, and is found in the C-terminal regions of surface-exposed Gram-negative bacterial proteins associated with autotransporters [16]. Proteins containing the YadA domain often have a characteristic functional organization, with an extended conserved N-terminal signal sequence (data not shown) and a central region that often includes Hep_Hag and HIM domains. In addition to the conserved domains, these proteins contain variable domains, which are often composed of low complexity sequence that are not conserved between family members. The domain conservation of these proteins has led us to designate them as *Burkholderia *Hep_Hag autotransporter (BuHA) proteins.

To define further the immunodominant region of these proteins, mapping was carried out to define the minimum region present in all positive clones at each of the four loci. For BMA0840, BMA1027 and BMAA1324, all of the clones contained Hep_Hag domains. For BMAA0649 all but one of the clones contained Hep_Hag domains, the errant clone contained the first 48 amino acids at the N-terminus. The Hep_Hag domain may therefore represent the minimum epitope required to stimulate an antibody response.

*In silico *analysis was conducted to define the presence of *B. mallei *BuHA proteins using the whole genome sequence of *B. mallei *ATCC 23344 [17]. Six genes were identified, four of which were the CDSs identified above (BMA0840, BMA1027, BMAA0649, and BMAA1324; Figure 1). Genes encoding the two non-immunogenic *B. mallei *BuHA proteins were examined. BMAA0810 contained a C-terminal YadA domain and a single HIM domain, but no Hep_Hag domains. The CDS encoding this protein had an IS element (IS407A) located immediately upstream and lacked an N-terminal signal sequence. IS element mediated recombination, a feature of the evolution of the *B. mallei *genome, appears to have resulted in the truncation of this CDS; it is likely that this CDS is a pseudogene. The other non-immunogenic member of this family is BMAA0749. This CDS contains Hep_Hag and HIM domains (Figure 1), but lacks a strong Pfam YadA domain match in the C-terminus; a weaker match to the YadA domain with a score below the Pfam gathering threshold (score 21.10, e-value 7.4e-06) was detected. This CDS also lacked an N-terminal signal sequence, and it is likely that the product is not processed to the external surface of the cell. These observations could explain the failure to detect these two loci by immuno-screening.

Six *B. mallei *isolates are currently undergoing whole genome sequencing by TIGR [18], and are publicly available via the Pathema database [19]. Alleles of all four immunodominant proteins were identified in strains 10399, FMN, JHU, and GB8; the number of residues in the encoded proteins was also comparable. Strain GB8 is a horse passaged derivative of ATCC 23344, and strains FMN and JHU are human passaged derivatives of ATCC 23344 isolated during the same infective episode. Two of the strains, NCTC 10229 and NCTC 10247, did not contain alleles of BMA1027. Additionally, both of these strains contained identical alleles of BMAA0649, which encoded proteins with additional internal residues (172 amino acids) in comparison to the ATCC 23344 protein. NCTC 10229 was originally isolated in Hungary in 1961, and NCTC 10247 was isolated in Turkey in 1960. Given their different origins, it is surprising that they both lack BMA1027 and have identical variants of BMAA049.

The distribution of the four immunogenic *B. mallei *BuHA proteins was investigated in collections of *B. mallei *strains (n = 21) using PCR. All isolates were positive for BMA0840 and BMAA1324, whilst BMA1027 and BMAA0649 showed some variability: 12/21 (57%) of strains were positive for BMA1027 and 18/21 (86%) of strains were positive for BMAA0649. The screening was expanded to include a collection of *B. pseudomallei *strains (n = 100). All isolates were positive for the *B. pseudomallei *homologs of BMA0840 and BMAA1324, while homologs of BMA1027 and BMAA0649 were variable but more widely distributed; 96/100 (96%) and 92/100 (92%) of the strains were positive for BMA1027 and BMAA0649, respectively.

### Distribution of BuHA proteins

We hypothesized that the BuHA proteins are important virulence determinants in *B. mallei *and *B. pseudomallei*, and that these would not be present in the highly related but non-pathogenic *B. thailandensis *genome [20]. *In silico *analysis using the whole genome sequences of *B. mallei *ATCC 23344 [17], *B. pseudomallei *K96243 [21] and *B. thailandensis *strain E264 [20] was performed to identify orthologs of the *B. mallei *BuHA proteins.

**Figure 2:**
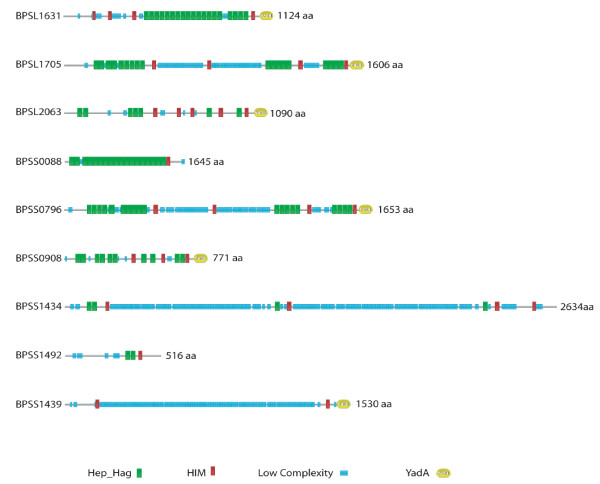
**Domain architecture of *Burkholderia pseudomallei *BuHA proteins**. Pfam [36] domain organization of BuHA proteins from *B. pseudomallei*. Keys are shown for the domains in the bead diagrams.

The genome of *B. pseudomallei *K96243 contained 9 genes encoding BuHA proteins (Figure 2). This included orthologs of all four *B. mallei *immunodominant proteins and the other two non-immunogenic proteins (Table 2). With the exception of the *B. pseudomallei *proteins BPSL1631 and BPSS0796, all of the orthologs contained a similar number of amino acid residues and were of a similar size. In the case of BPSL1631 and BPSS0796, there were increased residues in the repeat regions of these CDSs; BPSL1631 contained an additional 113 amino acid residues that corresponded to 4 Hep_Hag domains (Figures 1 and 2); and BPSS0796 contained an additional 118 amino acid residues in a low complexity region (Figures 1 and 2). The *B. pseudomallei *ortholog of BMAA0810, BPSS1439, is not truncated and contains an N-terminal signal sequence, HIM domains, and a C-terminal YadA domain, but does not contain Hep_Hag domains.

In addition to the orthologs of the *B. mallei *BuHA proteins, the *B. pseudomallei *genome contained three other related CDSs: BPSL1705, BPSS0088 and BPSS1434. All of these proteins contain HIM, Hep_Hag and YadA domains; the YadA domain matches for BPSS0088 and BPSS1434 are below the Pfam gathering threshold (score 15.2, e-value 2.1e-05 and score 21.8, e-value 4.8e-06 respectively).

The genome of *B. thailandensis *strain E264 contained 6 BuHA proteins (Table 2): 4 with Hep-Hag and YadA domains (BTH_II0112, BTH_II0878, BTH_II0957 and BTH_II1489); 1 protein with Hep-Hag domains but lacking a C-terminal domain (BTH_II0875); and 1 gene remnant (BTH_II0954) of the *B. mallei *BMAA0810 and *B. pseudomallei *BPSS1439 orthologs. Only one of these proteins was orthologous to the *B. mallei *immunodominant proteins (Table 2); two were orthologous to the non-immunogenic proteins, two have *B. pseudomallei *orthologs, but not *B. mallei *orthologs, and one is unique to *B. thailandensis*.

**Figure 3:**
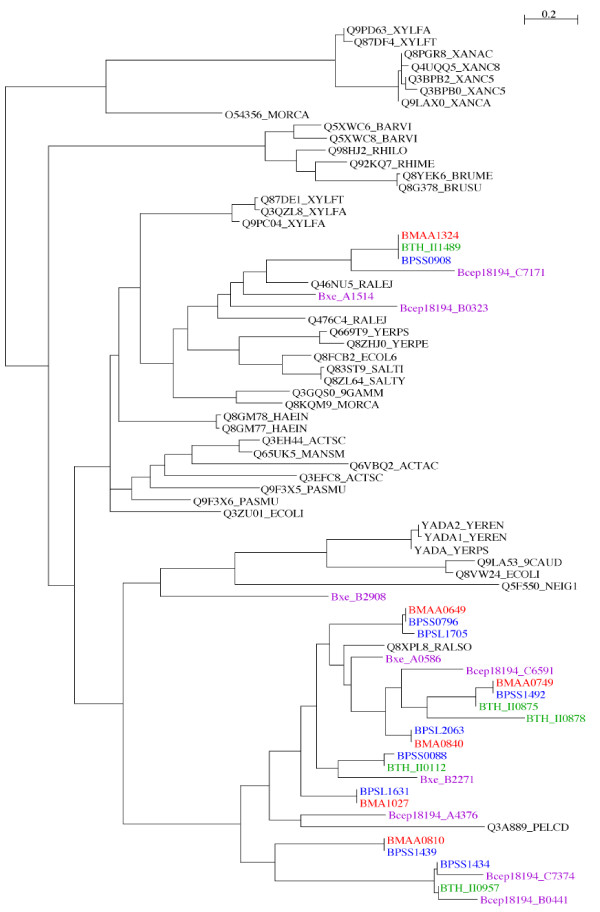
**Phylogenetic relationships of BuHA proteins based on C-terminal YadA domains**. Unrooted Maximum likelihood tree built from an alignment of YadA domain sequences from Hep_Hag family proteins. YadA domain sequences were extracted from *B. mallei *ATCC 23344,* B. pseudomallei *K96243,* B. thailandensis *E264, *B. xenovorans *LB400, *and Burkholderia *sp. 383 genome annotations; additional sequences were downloaded from the Pfam website [36,46]. *B. mallei *proteins are coloured red, *B. pseudomallei *proteins are coloured blue, *B. thailandensis *proteins are coloured green, other *Burkholderia *species proteins are coloured purple.

The identification of numerous hemagglutinin family proteins in the three *Burkholderia *genomes is in marked contrast to other bacterial species, including some other members of the Burkholderiacae which have lower numbers. The phylogenetic relationship of this family of proteins was investigated by aligning the C-terminal YadA domain regions. The repetitive distribution of Hep_Hag domains within proteins, and the variability and low complexity composition of other regions, renders whole protein comparisons unsuitable for phylogenetic analysis. The BuHA proteins from *B. mallei*, *B. pseudomallei*, *B. thailandensis *and 2 other sequenced *Burkholderia *species (*Burkholderia xenovorans *and *Burkholderia *sp. 383), were compared with BuHA proteins from other bacteria (Figure 3). The genomes of other Burkholderiaceae contained varying number of BuHA proteins (*Burkholderia *sp. 383 contains 6; *Burkholderia xenovorans *LB400 contains 4; and *Ralstonia solanacearum *GMI1000 contains 1). The BuHA proteins from *B. mallei *and *B. pseudomallei *were represented in two lineages. The *B. mallei *protein BMAA1324 clustered with orthologs from *B. pseudomallei *and *B. thailandensis *(Table 2) and a protein from *Burkholderia *sp. 383, and formed part of a larger cluster that was made up predominantly of proteins from outside the Burkholderiaceae. The other BuHA proteins from *B. mallei*, *B. pseudomallei *and *B. thailandensis *resided within a second cluster.

The orthogous relationships identified for the *B. mallei *and *B. pseudomallei *proteins using comparative genomic analysis were also evident in the phylogenetic tree (Figure 3). In addition, the tree topology suggested paralogous relationships for some of the BuHA proteins. For example, the orthologous pair *B. pseudomallei *protein BPSS0796 and *B. mallei *protein BMAA0649 clustered with *B. pseudomallei *protein BPSL1705. BPSL1705 resides in a genomic island (GI 8) [21] on chromosome I and BPSS0796 resides on chromosome II, but phylogenetic analysis of the YadA domains suggests that the two loci arose from a duplication event. The conservation of Hep_Hag and HIM domains organization in these two proteins also suggests a common ancestry. On this basis, it is possible that mobile genetic elements may have had a role in the expansion of BuHA proteins in *B. pseudomallei*.

### *B. pseudomallei *expression library screening with melioidosis patient serum

The finding that *B. pseudomallei *K96243 contains 9 genes encoding BuHA proteins raised the possibility that these may also be immunogenic during human melioidosis. A *B. pseudomallei *K96243 expression library was probed with twenty-one separate serum samples from patients presenting to Sappasithiprasong Hospital in northeast Thailand with culture-proven melioidosis. Patient age ranged from 10–71 years (median 49 years, IQR 31–56 years), and 15 were male. Diabetes mellitus was present in 12 patients, a proportion that reflects the presence of this condition in the larger melioidosis population. Manifestations of disease were highly variable, as follows: 7 patients had disseminated melioidosis (blood culture positive plus >1 non-contiguous foci of infection), 4 patients had bacteremia with a single or no identifiable focus of infection, 2 patients had multifocal infection (>1 non-contiguous foci of infection and blood culture negative), and 8 patients had localized disease (single focus of infection and blood culture negative).

A total of 189 clones, corresponding to 131 different loci (comprising a contiguous DNA region from single or overlapping clones) containing 428 known or putative open reading frames of *B. pseudomallei *were isolated. The BuHA proteins were uncommon; 5 of the putative *B. pseudomallei *BuHA proteins were present in either one (BPSL2063, BPSS0908, BPSS0796 and BPSS1439), or two clones (BPSS1492) (total 6/189 (3%) of clones overall); the other members of the BuHA protein group were not observed. Three of the four *B. mallei *homologs were each represented once.

## Discussions and conclusion

The findings of this study indicate that the BuHA proteins generate a strong antibody response in the experimental equine model of glanders. Bacteriophage-mediated immunoscreening demonstrated that four BuHA proteins were highly over-represented. Comparative sequence analysis of the cloned inserts of these four loci identified the minimum regions present in all clones. For each protein the mapped regions contained Hep_Hag motifs. We propose that this domain represents the immunostimulatory region of these proteins; the presence of multiple Hep_Hag protein domains may indicate that there are multiple sites for immune recognition. *In silico *analysis of the *B. mallei *ATCC 23344 genome identified two further members of this protein family (BMAA0749 and BMAA0810); their absence in the library can be explained by the fact that these were predicted not to be expressed or processed correctly.

In addition to the Hep_Hag domain, the four *B. mallei *immunostimulatory BuHA proteins contain C-terminal YadA trimeric autotransporter domains. This domain is found in a large family of surface proteins, and has been shown to be important in protein processing and transport. The YadA domain inserts into the outer membrane forming a trimeric structure, which then translocates the passenger domains to the cell surface side of the membrane [16]. Unlike other autotransporter proteins (for example, proteins containing the Pfam domain PF03797), the passenger domains of YadA domain proteins are not cleaved and remain covalently linked to the translocator domain. Stable trimerization has been shown to be essential for native folding and stability of the functional passenger domain [22]. Several of the YadA family proteins have been functionally characterized and shown to be adhesins that mediate bacterial interactions with host cells or extracellular matrix proteins [23-28]. The prototypical protein of this family is the YadA adhesin from *Yersinia enterocolitica *[29]. In addition to the C-terminal YadA domain, the protein also contains 4 Hep_Hag domains and 1 HIM domain. YadA has a trimeric head-stalk-anchor architecture [30], with the Hep_Hag and HIM domains forming part of the head domain [31] (Figure 1). This region of the protein has a left-handed parallel β-roll (LPBR) structure, which has been shown to bind collagen [31]. In particular, residues within the Hep_Hag domain regions of YadA have been shown to be essential for YadA-mediated collagen binding [32]. Epitope mapping of YadA identified 7 epitopes that were uniquely recognized by an anti-YadA antiserum that was able to inhibit collagen binding [32]. Four of these epitopes shared a motif NSVAIG-S that is repeated eight times within the N-terminal half of YadA and forms part of the Hep_Hag motif.

The role of *B. mallei *immunostimulatory BuHA proteins is unknown, but the similarity in domain organisation to adhesins such as YadA suggests they may function as cell surface binding proteins that potentially modulate host-cell interactions. The redundancy of proteins in the genomes, and the diversity of the proteins, suggests that they may have functionally distinct roles. Although the Hep_Hag domain of the YadA protein of *Y. enterocolitica *has been shown to bind collagen, it is not known if any of the *B. mallei *proteins are able to bind this host molecule. Further work is needed to characterize the functions and specificities of these proteins, and the role they play in the pathogenesis of *B. mallei*.

Orthologues of all the *B. mallei *BuHA proteins were identified in the *B. pseudomallei *K96243 genome, together with three further CDSs (BPSL1705, BPSS0088 and BPSS1434). These additional CDSs were in regions of the *B. mallei *ATCC23344 genome that appear to have been deleted in comparison to the *B. pseudomalllei *K96243 genome [21]. However, this family of proteins was not strongly immunostimulatory during human melioidosis, despite evidence for expression *in vivo *as indicated by a small number of positive clones in the library. Possible explanations for the difference in immunostimulatory capacity of *B. mallei *in horses and *B. pseudomallei *in humans includes variation in the nature of the immune response or other aspects of disease pathogenesis in the horse versus human host, and different rates of gene regulation and expression between the two bacterial species. It is also possible that protein folding and associated immunogenicity differs between the two bacterial species, although this seems less tenable given their degree of relatedness [33].

The implications of these findings for vaccine development and diagnostics are currently uncertain. It is unclear whether the BuHA proteins are immunogenic during human *B. mallei *infection. Given the relative lack of immune response to the BuHA proteins during human melioidosis, it seems unlikely that detection of antibodies to BuHA proteins will have diagnostic utility for this infection. Although there are apparent differences in the immunostimulatory profiles of the hemaglutinin family proteins of *B. mallei *and *B. pseudomallei *in their respective disease model, both pathogens share an expanded number of these proteins in comparison to the non-pathogenic *B. thailandensis*. Both *B. pseudomallei *and *B. thailandensis *can be isolated from the soil. We speculate on the basis of a reduced number of hemaglutinin family proteins othologues in *B. thailandensis *that they may have a role in modulating host-cell interactions in *B. pseudomallei*. Further studies will be required to investigate the *in vivo *expression of these proteins in the host, and the role they play in the pathology of melioidosis.

## Methods

### Bacterial strains

*E. coli *strains were grown in Luria-Bertani medium, using selection with kanamycin (50 μg/ml), where appropriate. *B. pseudomallei *and *B. mallei *were grown in TSB or TSB plus 4% glycerol, respectively.

### Serum

Sera taken from two separate horses obtained 7 days after intra-tracheal inoculation of a suspension containing *B. mallei *ATCC 23344 together with a control serum from a horse pre-inoculation were kindly provided by Donald E Woods, Faculty of Medicine, University of Calgary. Sera from 21 patients with culture-confirmed melioidosis were collected on admission to Sappasithiprasong Hospital, Ubon Ratchathani, northeast Thailand between 1998 and 2003. Attributable days of symptoms prior to presentation ranged from 3 to 150 days (median 14 days, inter-quartile range (IQR) 7 to 28 days).

### Screening of *B. mallei *and *B. pseudomallei *expression libraries with equine or human sera

Bacteriophage were propagated on *E. coli *XL1-Blue MRF' and plaque lifted onto Immobilon-NC nitrocellulose membranes (Millipore) pre-soaked in 10 mM IPTG, according to the manufacturer's instructions. After overnight blocking at 4°C in TBST (10 mM Tris-HCl [pH 7.4], 0.15 M NaCl, 0.05% [vol/vol] Tween 20) containing 6% (wt/vol) skimmed milk powder, hybridization was carried out with equine or human sera diluted 1:1,000 (in TBST-milk) for 90 min at room temperature. Blots were then incubated with alkaline phosphatase-conjugated anti-human or anti-equine IgG gamma chain specific monoclonal antibodies (Sigma) diluted 1:50,000 (in TBST) for 30 min at room temperature. Bound antibodies were detected using nitroblue tetrazolium (NBT)-BCIP (5-Bromo-4-chloro-3-indolylphosphate) solution (Roche). After a second purification round of screening, phagemids containing cloned *B. mallei *or *B. pseudomallei *DNA were excised into *E. coli *XLOLR according to the manufacturer's instructions.

Excised phagemid DNA was purified using QIAprep spin miniprep columns (QIAGEN) and the *B. pseudomallei *or *B. mallei *insert DNA sequenced using a DYEnamic ET Dye Terminator Kit (Amersham) and MegaBACE 500 sequencer. BLASTN searches using sequences from either end of the insert were carried out against the *B. mallei *ATCC 23344 genome [17,18], or the *B. pseudomallei *K96243 genome [21,34]. Mapping of positive clones were performed using Artemis software [35]. Domain organization of proteins was defined and compared using the Pfam [15] website [36].

### PCR detection of genes encoding BuHA proteins in *B. mallei *and *B. pseudomallei*

Two bacterial strain collections were examined; 21 *B. mallei *isolates have been described previously [37], and 100 *B. pseudomallei *isolates were from patients with melioidosis presenting to Sappasithiprasong Hospital during 2001 (n = 50) or recovered from the environment in northeast Thailand (n = 50). A single colony picked from solid agar was inoculated into TSB and incubated overnight in air at 37°C, after which genomic DNA was extracted using the Wizard Genomic DNA purification kit (Promega).

The presence or absence of four BuHA proteins in *B. mallei *and their homologs in *B. pseudomallei *was defined by PCR. The primers used were as follows: BMA0840-f (5'-AGCGGCGCGGGGTCCTATTC), BMA0840-r (5'-CCGCCGCCACGTTGATGAG); BMA1027-f (5'-AGCGCGGGCAGCATTTACTTCC), BMA1027-r (5'-GATTCGCGTTGACCGTGCTGAGG); BMAA0649-f (5'-CGGTGAACGGCTCGCAGATGAATG), BMAA0649-r (5'-GGCCCCGCGAACCGAACGACAC); and BMAA1324-f (5'-GCCGCGGCGCAGGTCAG), BMAA1324-r (5'-CCGTCGCCCGCCGCTTCC).

The final concentrations of the PCR mixtures were 1X reaction buffer, 1.5 mM MgCl_2_, 0.35 μM of each primer, 5 μl of 1:20 dilution template DNA, 200 μM dNTPs and 2.5 unit Taq polymerase (Promega). Samples were held at 95°C for 2 min and then subjected to 40 cycles of 95°C for 30 s, 68°C (BMA0840 & BMA1027) or 70°C (BMAA0649 & BMAA1324) for 30 s, and 72°C for 60 s, followed by a final extension step of 72°C for 5 min. Reactions for BMA 0840 and BMA 1027 were multiplexed. PCR amplifications were performed using a PTC-0200 DNA engine (MJ Research, Cambridge, MA), and aliquots of reaction mixtures were analyzed by 2% agarose gel electrophoresis.

### *In silico *identification of orthologs of BuHA proteins

Orthologs of BuHA proteins in the genomes of *B. pseudomallei *K96243 (accession numbers BX571965 and BX571966) [21], *B. mallei *ATCC 23344 (accession numbers CP000010 and CP000011) [17] and *B. thailandensis *E264 (accession numbers CP000086 and CP000085) [20] was carried out by reciprocal FASTA [38] analysis as previously described [39]. Coding sequences containing Hep_Hag (PF05658) and YadA (PF03895) domains were identified using HMMER [40] and the respective Pfam profile HMMs downloaded from the Pfam website [36]. The identification of alleles of BuHA proteins in 6 *B. mallei *isolates (10399, FMN, JHU, GB8, 10229 and NCTC10247) currently undergoing whole genome sequencing by The Institute of Genome Research (TIGR) [18] was carried out using BLASTN [41]. Additional Burkholderiaceae genomes sequences used in this study were from: *Burkholderia *sp. 383 (accession numbers CP000150, CP000151 and CP000152), *Burkholderia xenovorans *LB400 (accession numbers CP000270, CP000271 and CP000272) and *Ralstonia solanacearum *GMI1000 (accession numbers AL646052 and AL646053) [42].

### Phylogenetic analysis

An unrooted maximum likelihood tree built using Phylip (Version 3.6) [43] was drawn using NJplot [44]. Sequences were aligned using ClustalX (Version 1.82) [45].

## Authors' contributions

RT carried out the *in vivo *gene expression component, MTGH carried out the bioinformatics component and drafted the manuscript, ST carried out the *in vivo *gene expression component, SR contributed critical review, SRC contributed to the experimental concept and design and provided technical support, SJF contributed to the experimental concept and design and provided technical support, WCN assisted with the bioinformatics component and assisted with drafting the manuscript, NPJD contributed towards study design and undertook critical review of the manuscript, and SJP conceived of the study, participated in the study design and coordination, and drafted the manuscript. All authors read and approved the final manuscript.

## Supplementary Material

Additional file 1 Loci identified on immunoscreening of the *B. mallei *library. Click here for file

## Figures and Tables

**Table 1:** The four most frequent loci identified on immunoscreening of the *B. mallei *library.

**Locus**	**Locus co-ordinates**	**Clone co-ordinates**	**Clone occurrence**	**Genes within locus (with co-ordinates)**	**Name, putative function of homology**
10	875663–881753	875663–880118	1	BMA0838 (875959–876654)	DNA-binding response regulator
		876370–881176	1	BMA0839 (876702–876944)	Hypothetical protein
		876765–880560	2	BMA0840 (877030–880302)	Haemagglutinin family protein
		876928–880933	1	BMA0841 (880422–881093)	OmpA family protein
		876928–880678	3	BMA0842 (881095–882624)	TPR Domain domain protein
		877235–881753	1		
		877402–880118	1		
		877579–880118	2		
		877579–879740	3		
		877235–880659	3		
		877575–880933	1		
		877581–880663	2		
		878122–880678	1		
		879170–880659	1		
		879216–880873	1		

14	1072256–1078767	1072256–1076403	1	BMA1024 (1072412–1073026)	Type-1 fimbrial protein, A subunit
		1072709–1077699	1	BMA1025 (1073192–1073653)	Pseudogene
		1072731–1076799	2	BMA1026 (1073869–1074054)	Hypothetical protein
		1073129–1078023	2	BMA1027 (1074198–1077236)	Outer membrane protein, putative
		1073358–1076799	3	BMA1028 (1077244–1077492)	Hypothetical protein
		1073358–1078533	1	BMA1029 (1077515–1077694)	Pseudogene
		1073625–1076884	3	BMA1030 (1077715–1077978)	Pseudogene
		1074020–1076884	2	BMA1031 (1077953–1078135)	Pseudogene
		1074281–1076800	1	BMA1032 (1078196–1078453)	Pseudogene
		1074570–1076800	2	BMA1033 (1078422–1079098)	Pseudogene
		1074570–1076992	8		
		1074570–1077699	1		
		1074570–1077970	2		
		1074598–1076992	1		
		1074858–1076799	1		
		1075270–1078767	1		
		1075270–1076799	1		
		1075414–1077699	1		
		1075414–1078023	1		
		1075414–1078078	2		
		1075414–1078309	1		
		1075421–1077970	1		
		1075438–1077970	1		

54	657988–664905	657988–661537	2	BMAA0649 (658368–662975)	Hep_Hag family protein/haemagglutinin motif family
		657991–661537	3		protein/YadA-like domain protein
		658070–660535	1	BMAA0650 (658368–662975)	Hypothetical protein
		658080–661537	1	BMAA0651 (663846–664187)	H-NS histone family protein
		658345–660890	1	BMAA0652 (664378–665118)	Transcriptional regulator, IclR family
		658485–663240	1		
		658485–660037	1		
		659301–663317	1		
		659301–663508	2		
		659440–661453	1		
		659519–661453	1		
		659675–663508	1		
		659947–663508	1		
		660568–662869	1		
		660906–663317	1		
		660906–663909	2		
		661040–663303	2		
		661040–664905	1		

61	1422362–1427764	1422362–1425667	1	BMAA1320 (1421605–1423098)	Amidase family protein
		1422767–1427459	1	BMAA1321 (1423230–1423490)	Hypothetical protein
		1424023–1426561	1	BMAA1322 (1423632–1424066)	UspA family protein
		1424255–1427209	3	BMAA1323 (1424227–1425042)	Outer membrane protein OmpA/SmpA/OmlA family
		1424255–1427211	1	BMAA1324 (1425116–1427611)	Haemagglutinin family protein
		1424263–1427203	1		
		1424846–1426767	2		
		1424886–1426776	1		
		1424846–1426977	1		
		1424846–1427211	1		
		1424846–1427764	2		
		1425017–1427764	2		
		1425067–1427764	1		
		1425768–1426773	2		
		1425774–1426766	2		

**Table 2:** Orthologs of BuHA proteins in Burkholderia pseudomallei, *Burkholderia mallei and Burkholderia thailandensis*.

*B. mallei*	*B. pseudomallei*	*B. thailandensis*
BMA0840	BPSL2063	-
BMA1027	BPSL1631	-
-	BPSL1705	-
BMAA0649	BPSS0796	-
BMAA1324	BPSS0908	BTH_II1489
BMAA0810*§	BPSS1439§	BTH_II0954*§+
BMAA0749+	BPSS1492+	BTH_II0875+
-	BPSS1434+	BTH_II0957
-	BPSS0088+	BTH_II0112
-	-	BTH_II0878+

Orthologs of BuHA proteins identified in the complete genomes of *B. pseudomallei *K96243, *B. mallei *ATCC 23344 and *B. thailandensis *E264. The presence of a BuHA protein is indicated by the systematic identifier for that protein in the genome, – indicates absence of an orthologue. Orthology was determined by viewing pairwise BLASTN [41] comparisons of the genomes in ACT [47] to identify loci displayed similarity and conserved surrounding gene order. * Pseudogene, §Below Pfam trusted threshold Hep_Hag domain match, + Below Pfam trusted threshold or no YadA domain match.
