# Supplementary material for: Burkholderia Hep_Hap autotransporter (BuHA) proteins elicit a strong antibody response during experimental glanders but not human melioidosis
Source: BMC Microbiol. 2007 Mar 15;7:19. doi: 10.1186/1471-2180-7-19 (PMC1847439; doi:10.1186/1471-2180-7-19)
Supplement: Additional file 1 — Loci identified on immunoscreening of the B. mallei library. [file 1471-2180-7-19-S1.doc]

| **Locus** | **Locus co-ordinates** | **Clone**  **co-ordinates** | **Clone occurrence** | **Genes within locus (with co-ordinates)** | **Name, putative function of homology** |
| --- | --- | --- | --- | --- | --- |
| 1 | 157964-160506 | 157964-160506  157970-160506 | 1  1 | BMA0146 (156746-158155)  BMA0147 (158152-158742)  BMA0148 (158732-161152) | Sun protein  Hypothetical protein  Nitrogen regulation protein NtrY, putative |
| 2 | 178706-182206 | 178706-182206 | 2 | BMA0168 (178805-180260)  BMA0169 (180457-180639)  BMA0170 (180663-181067)  BMA0171 (181159-182124)  BMA0172 (182094-183008) | ISBma2, transposase  Hypothetical protein  hypothetical protein  Esterase, putative  hypothetical protein |
| 3 | 317596-321682 | 317596-320859  319125-321682 | 2  1 | BMA0302 (316984-318180)  BMA0303 (318192-319217)  BMA0304 (319471-320610)  BMA0305 (320715-321632) | Phosphoribosylaminoimidazole carboxylase  Sua5/YciO/YrdC/YwlC family protein  Hypothetical protein  Pseudogene |
| 4 | 411831-413839 | 411831-413839 | 1 | BMA0387 (412143-412646)  BMA0388 (412775-414238) | Dihydrofolate reductase  ISBma2, transposase |
| 5 | 461820-463848 | 461820-463848 | 1 | BMA0435 (459302-461902)  BMA0436 (462413-463087)  BMA0437 (463256-463954) | DNA gyrase subunit A  OmpA family protein  3-demethylubiquinone-9 3-methyltransferase |
| 6 | 552397-556679 | 552397-556679 | 1 | BMA0524 (551691-552692)  BMA0525 (552731-553453)  BMA0526 (553450-554097)  BMA0527 (554341-554973)  BMA0528 (555070-555249)  BMA0529 (555388-556494)  BMA0530 (556494-557483) | Peptidase, U7 family protein  tetrapyrrole methylase family protein  Maf-like protein  Hypothetical protein  50S ribosomal protein L32  Fatty acid/phospholipid synthesis protein  3-oxoacyl-(acyl-carrier-protein) synthase |
| 7 | 598774-601540 | 598774-601540 | 1 | BMA0573 (599509-600342)  BMA0574 (600366-600629)  BMA0575 (600776-601822) | IS407A, transposase OrfB  IS407A, transposase OrfA  Hypothetical protein |
| 8 | 736697-737872 | 736697-737872 | 1 | BMA0710 (736321-736707)  BMA0711 (736677-737324)  BMA0712 (737706-738794) | Hypothetical protein  OmpA family protein  ParA family protein |
| 9 | 764575-766940 | 764575-766940 | 2 | BMA0733 (764095-765402)  BMA0734 (765441-766199)  BMA0735 (766288-767727) | Major facilitator family protein  3-ketoacyl-(acyl-carrier-protein) reductase  aldehyde dehydrogenase family protein |
| 10 | 875663-881753 | 875663-880118  876370-881176  876765-880560  876928-880933  876928-880678  877235-881753  877402-880118  877579-880118  877579-879740  877235-880659  877575-880933  877581-880663  878122-880678  879170-880659  879216-880873 | 1  1  2  1  3  1  1  2  3  3  1  2  1  1  1 | BMA0838 (875959-876654)  BMA0839 (876702-876944) BMA0840 (877030-880302) BMA0841 (880422-881093)  BMA0842 (881095-882624) | DNA-binding response regulator  Hypothetical protein  Hemagglutinin family protein  OmpA family protein TPR Domain domain protein |
| 11 | 911916-914717 | 911916-914717 | 1 | BMA0877 (912753-913694)  BMA0878 (914222-916186) | Conserved hypothetical protein  Sensor histidine kinase/response regulator |
| 12 | 959381-961327 | 959381-961327 | 1 | BMA0914 (958630-959430)  BMA0915 (959443-960567)  BMA0916 (960689-961738) | Sugar ABC transporter, ATP-binding protein, putative  Sugar ABC transporter, permease protein, putative  Sugar ABC transporter, periplasmic sugar-binding protein, putative |
| 13 | 1009471-1012843 | 1009471-1012843 | 1 | BMA0956 (1008670-1010409)  BMA0957 (1010519-1011589)  BMA0958 (1011700-1012593)  BMA0959 (1012655-1013140) | Putative voltage-gated ClC-type chloride channel  Hypothetical protein  Transcriptional regulator, LysR family  Hypothetical protein |
| 14 | 1072256-1078767 | 1072256-1076403  1072709-1077699  1072731-1076799  1073129-1078023  1073358-1076799  1073358-1078533  1073625-1076884  1074020-1076884  1074281-1076800  1074570-1076800  1074570-1076992  1074570-1077699  1074570-1077970  1074598-1076992  1074858-1076799  1075270-1078767  1075270-1076799  1075414-1077699  1075414-1078023  1075414-1078078  1075414-1078309  1075421-1077970  1075438-1077970 | 1  1  2  2  3  1  3  2  1  2  8  1  2  1  1  1  1  1  1  2  1  1  1 | BMA1024 (1072412-1073026)  BMA1025 (1073192-1073653)  BMA1026 (1073869-1074054) BMA1027 (1074198-1077236)BMA1028 (1077244-1077492)BMA1029 (1077515-1077694) BMA1030 (1077715-1077978)  BMA1031 (1077953-1078135)  BMA1032 (1078196-1078453)  BMA1033 (1078422-1079098) | Type-1 fimbrial protein, A subunit  Pseudogene  Hypothetical protein Outer membrane protein, putative Hypothetical protein  Pseudogene  Pseudogene  Pseudogene  Pseudogene  Pseudogene |
| 15 | 1109324-1112596 | 1109324-1112596  1110278-1112596  1110370-1112596 | 2  1  1 | BMA1061 (1109405-1112332)  BMA1062 (1112424-1113899) | Translation initiation factor IF-2, infB,  Transcription elongation factor NusA |
| 16 | 1134323-1136703 | 1134323-1136703 | 1 | BMA1091 (1133630-1136062)  BMA1092 (1136150-1137163) | Phenylalanyl-tRNA synthetase, beta subunit  Phenylalanyl-tRNA synthetase, alpha subunit |
| 17 | 1162254-1165249 | 1162254-1165249 | 1 | BMA1121 (1162443-1163504)  BMA1122 (1164495-1165112) | JmjC domain protein  Hypothetical protein |
| 18 | 1318906-1321276 | 1318906-1321276 | 1 | BMA1267 (1317496-1319682)  BMA1268 (1319710-1321230) | Methyl-accepting chemotaxis domain protein  Phosphoesterase family protein |
| 19 | 1395999-1397980 | 1395999-1397980 | 1 | BMA1337 (1395779-1396678)  BMA1338 (1396695-1398008) | ftsH protease activity modulator HflC  ftsH protease activity modulator HflK |
| 20 | 1624672-1627170 | 1624672-1627170 | 1 | BMA1563 (1623708-1627220) | Chromosome segregation protein SMC |
| 21 | 1686221-1688184 | 1686221-1688184 | 1 | BMA1623 (1686349-1687314)  BMA1624 (1687470-1688288) | Sulfate adenylyltransferase, subunit 2, cysD-2  Glycosyl transferase, group 2 family protein |
| 22 | 1809051-1811501 | 1809051-1811501 | 1 | BMA1729 (1808888-1810117)  BMA1730 (1810161-1811546) | Nitrate/nitrite transporter, NarD  Nitrate/nitrite transporter NarK |
| 23 | 1832465-1834908 | 1832465-1834908 | 2 | BMA1746 (1831927-1832760)  BMA1747 (1832816-1833088)  BMA1748 (1833304-1834293)  BMA1749 (1834310-1835302) | Hypothetical protein  Hypothetical protein  Pseudogene  Hypothetical protein |
| 24 | 1867413-1869705 | 1867413-1869705 | 1 | BMA1772 (1866300-1868576)  BMA1773 (1868573-1868764)  BMA1774 (1868761-1870125) | Hypothetical protein  Hypothetical protein  Amino acid permease |
| 25 | 1893093-1895858 | 1893093-1895858 | 1 | BMA1801 (1892595-1893749)  BMA1802 (1893980-1894612)  BMA1803 (1894876-1895667)  BMA1804 (1895677-1896390) | Putative aminotransferase  Glutathione S-transferase N-terminal domain protein  Enoyl-CoA hydratase  Phosphoglycerate mutase, putative |
| 26 | 2028439-2031525 | 2028439-2031525  2028859-2031105 | 1  1 | BMA1930 (2028321-2029271)  BMA1931 (2029276-2029920)  BMA1932 (2030053-2031123) | Ferredoxin  Endonuclease III, nth  Hypothetical protein |
| 27 | 2054855-2056857 | 2054855-2056857 | 1 | BMA1957 (2054252-2055034)  BMA1958 (2055116-2056012)  BMA1959 (2056027-2056716) | Hypothetical protein  Dihydrodipicolinate synthase, putative  MOSC domain protein |
| 28 | 2105036-2107295 | 2105036-2107295 | 1 | BMA2006 (2104178-2105185)  BMA2007 (2105209-2106123)  BMA2008 (2106108-2106674)  BMA2009 (2106668-2107183) | Alcohol dehydrogenase, zinc-containing protein  Hypothetical protein  Hypothetical protein  Activator protein, putative |
| 29 | 2144026-2145990 | 2144026-2145990 | 1 | BMA2046 (2144116-2144991)  BMA2047 (2145016-2146230) | Transcriptional regulator, AraC family  alanine--glyoxylate aminotransferase |
| 30 | 2165996-2169018 | 2165996-2169018 | 1 | BMA2067 (2165929-2166231)  BMA2068 (2166355-2166744)  BMA2069 (2166848-2167252)  BMA2070 (2167357-2167809)  BMA2071 (2168425-2169210) | Hypothetical protein  Hypothetical protein  Hypothetical protein  Type IV pilus biogenesis protein, putative  Hypothetical protein |
| 31 | 2176090-2178468 | 2176090-2178468 | 1 | BMA2080 (2175193-2176218)  BMA2081 (2176315-2177616)  BMA2082 (2177634-2178146)  BMA2083 (2178211-2178915) | Hypothetical protein  Translocation protein TolB precursor  Outer membrane protein, OmpA family  Hypothetical protein |
| 32 | 2196339-2199155 | 2196339-2198848  2196825-2199155 | 1  1 | BMA2106 (2195289-2196602)  BMA2107 (2196783-2196965)  BMA2108 (2197000-2198022)  BMA2109 (2198184-2199839) | Protein metabolite proton symporter family protein  Hypothetical protein  Lipopolysaccharide heptosyltransferase I, rfaC  Voltage gated chloride channel family protein |
| 33 | 2290949-2293768 | 2290949-2293768 | 1 | BMA2208 (2291111-2293093)  BMA2209 (2293152-2295470) | DNA topoisomerase IV subunit B, parE  DNA topoisomerase IV A subunit, parC |
| 34 | 2329112-2331133 | 2329112-2331133 | 1 | BMA2243 (2329016-2329516)  BMA2244 (2329578-2330789)  BMA2245 (2330853-2331299) | lipoprotein signal peptidase, lspA  phosphopantothenoylcysteine synthase/decarboxylase, coaBC  deoxyuridine 5`-triphosphate nucleotidohydrolase |
| 35 | 2352413-2356002 | 2352413-2356002  2353785-2356002 | 1  2 | BMA2267 (2352858-2354063)  BMA2268 (2354152-2354985)  BMA2269 (2355009-2355272)  BMA2270 (2355338-2356174) | Chromate transport protein  IS407A, transposase OrfB  IS407A, transposase OrfA  Pseudogene |
| 36 | 2399511-2401300 | 2399511-2401300 | 1 | BMA2307 (2398798-2399961)  BMA2308 (2400142-2401293) | Capsular polysaccharide biosynthesis/export periplasmic protein  Glycosyltransferase, putative |
| 37 | 2404745-2406434 | 2404745-2406434 | 1 | BMA2310 (2403707-2405134)  BMA2311 (2405405-2406241)  BMA2312 (2406307-2407179) | Mannose-1-phosphate guanylyltransferase/mannose-6-phosphate isomerase  yafJ protein, putative  Mechanosensitive ion channel YggB |
| 38 | 2508825-2511421 | 2508825-2511421 | 1 | BMA2403 (2508573-2509547)  BMA2404 (2509544-2510314)  BMA2405 (2510398-2511120)  BMA2406 (2511132-2512265) | Alkylphosphonate utilization operon protein PhnJ  Phosphonates ABC transporter, ATP-binding protein, PhnK  Pseudogene  Alkylphosphonate utilization operon protein, PhnM |
| 39 | 2515843-2520518 | 2515843-2520518 | 1 | BMA2410 (2515749-2516561)  BMA2411 (2516558-2517295)  BMA2412 (2517514-2518740)  BMA2413 (2518747-2519835)  BMA2414 (2519937-2521430) | Pyrroline-5-carboxylate reductase  Conserved hypothetical protein  Glycolate oxidase iron-sulfur subunit, GlcF  Glycolate oxidase, subunit GlcE  Glycolate oxidase, subunit GlcD |
| 40 | 2704159-2707101 | 2704159-2707101 | 1 | BMA2593 (2703584-2704414)  BMA2594 (2704550-2705356)  BMA2595 (2705367-2706200)  BMA2596 (2706224-2706487)  BMA2597 (2706685-2707875) | Outer membrane protein, OmpW family  Pseudogene  IS407A, transposase OrfB  IS407A, transposase OrfA  Cytochrome c assembly family protein |
| 41 | 2774166-2776782 | 2774166-2776782 | 1 | BMA2674 (2773416-2775449)  BMA2675 (2775904-2777031) | Type III DNA modification methyltransferase  Outer membrane porin, putative |
| 42 | 2817514-2820980 | 2817514-2820971  2817983-2820231  2818309-2820980 | 1  1  1 | BMA2729 (2817676-2817873)  BMA2730 (2817922-2819055)  BMA2731 (2819658-2821424) | Sulfur carrier protein, ThiS  Oxidoreductase, FAD-binding  ABC transporter, ATP-binding protein |
| 43 | 2845644-2849132 | 2845644-2849132  2846592-2849132 | 1  2 | BMA2752 (2845080-2847473)  BMA2753 (2847574-2849037) | Penicillin-binding protein, 1A family  ISBma2, transposase |
| 44 | 2853163-2855951 | 2853163-2855951 | 1 | BMA2759 (2853202-2854035)  BMA2760 (2854059-2854322)  BMA2761 (2854388-2854603)  BMA2762 (2854674-2855168)  BMA2763 (2855336-2855710)  BMA2764 (2855826-2856824) | IS407A, transposase OrfB  IS407A, transposase OrfA  flagellar biosynthetic protein FliP, interruption-N  flagellar protein FliO  flagellar motor switch protein FliN  flagellar motor switch protein FliM |
| 45 | 2966736-2968905 | 2966736-2968905 | 1 | BMA2874 (2966014-2967534)  BMA2875 (2967548-2967862)  BMA2876 (2967950-2970415) | Flagellar hook-associated protein 2  Hypothetical protein  TPR domain protein |
| 46 | 3006741-3008558 | 3006741-3008558 | 1 | BMA2915 (3006898-3007080)  BMA2916 (3007238-3008452)  BMA2917 (3008497-3009612) | Hypothetical protein  Sodium/hydrogen exchanger family protein  Hypothetical protein |
| 47 | 3113368-3115472 | 3113368-3115472 | 1 | BMA3010 (3112918-3114336)  BMA3011 (3114329-3114931)  BMA3012 (3115104-3116339) | Alpha, alpha-trehalose-phosphate synthase, otsA-2  Hypothetical protein  Hypothetical protein |
| 48 | 3401221-3405075 | 3401221-3404224  3402277-3404487  3402277-3405075 | 1  4  1 | BMA3290 (3400824-3402191)  BMA3291 (3402661-3404124)  BMA3292 (3404227-3404997) | DNA-binding response regulator, LuxR family  ISBma2, transposase  Ferredoxin--NADP reductase, fpr-2 |
| 49 | 3443217-3446275 | 3443217-3446275 | 1 | BMA3334 (3442796-3443554)  BMA3335 (3443760-3445763)  BMA3336 (3445779-3447011) | Hypothetical protein  Flagellar hook-associated protein, flgK  Flagellar hook-associated protein 3, flgL |
| 50 | 3501070-3502346 | 3501070-3502346 | 1 | BMA3390 (3500829-3501869)  BMA3391 (3502208-3502777) | Sodium/bile acid symporter family protein  Hypothetical protein |
| 51 | 595666-599070 | 595666-598656  596521-599070  596806-599070 | 2  1  1 | BMAA0587 (594954-596051)  BMAA0588 (596346-596609)  BMAA0589 (596670-597041)  BMAA0590 (597208-598008)  BMAA0591 (598130-599539) | Putative outer membrane porin  Hypothetical protein  Hypothetical protein  Transcriptional regulator, LuxR family  Amino acid permease |
| 52 | 604189-606205 | 604189-606205 | 1 | BMAA0595 (602868-605105)  BMAA0596 (605316-606326) | Fusaric acid resistance domain protein  Transcriptional regulator, LysR family |
| 53 | 620307-623320 | 620307-623320 | 1 | BMAA0610 (620338-621948)  BMAA0611 (621952-623619) | di-haem cytochrome c peroxidase family protein  Phosphoesterase family protein |
| 54 | 657988-664905 | 657988-661537  657991-661537  658070-660535  658080-661537  658345-660890  658485-663240  658485-660037  659301-663317  659301-663508  659440-661453  659519-661453  659675-663508  659947-663508  660568-662869  660906-663317  660906-663909  661040-663303  661040-664905 | 2 3  1  1  1  1  1  1  2  1  1  1  1  1  1  2  2  1 | BMAA0649 (658368-662975)BMAA0650 (658368-662975)BMAA0651 (663846-664187)BMAA0652 (664378-665118) | Hep_Hag family protein/hemagglutinin motif family protein/YadA-like domain protein  Hypothetical protein  H-NS histone family protein  Transcriptional regulator, IclR family |
| 55 | 792810-795028 | 792810-795028 | 1 | BMAA0773 (794478-795152) | Transcriptional regulator, TetR family |
| 56 | 944471-946610 | 944471-946610 | 1 | BMAA0925 (944052-945305)  BMAA0926 (945792-946001)  BMAA0927 (945965-948163) | Multidrug resistance protein  Hypothetical protein  Prolyl oligopeptidase family protein |
| 57 | 966275-968707 | 966275-968671  966275-968707 | 1  1 | BMAA0950 (966184-968439) | Major facilitator family transporter |
| 58 | 1021073-1022928 | 1021073-1022928 | 1 | BMAA0994 (1021079-1021759)  BMAA0995 (1021927-1022445) | Hypothetical protein  Hypothetical protein |
| 59 | 1394221-1396115 | 1394221-1396115 | 1 | BMAA1290 (1394005-1394952)  BMAA1291 (1395568-1395966) | Ornithine cyclodeaminase  4-carboxymuconolactone decarboxylase |
| 60 | 1402166-1406263 | 1402166-1406212  1403258-1406212  1403504-1406212  1404127-1406263 | 1  1  1  1 | BMAA1297 (1403625-1403738)  BMAA1298 (1403739-1405331)  BMAA1299 (1405419-1406933) | Hypothetical protein  Methyl-accepting chemotaxis protein  Drug resistance transporter, EmrB/QacA family |
| 61 | 1422362-1427764 | 1422362-1425667  1422767-1427459  1424023-1426561  1424255-1427209  1424255-1427211  1424263-1427203  1424846-1426767  1424886-1426776  1424846-1426977  1424846-1427211  1424846-1427764  1425017-1427764  1425067-1427764  1425768-1426773  1425774-1426766 | 1  1  1  3  1  1  2  1  1  1  2  2  1  2  2 | BMAA1320 (1421605-1423098)  BMAA1321 (1423230-1423490)  BMAA1322 (1423632-1424066)  BMAA1323 (1424227-1425042)  BMAA1324 (1425116-1427611) | Amidase family protein  Hypothetical protein  UspA family protein  Outer membrane protein OmpA/SmpA/OmlA family  Hemagglutinin family protein |
| 62 | 1442087-1446422 | 1442087-1446422  1444517-1446422 | 1  1 | BMAA1339 (1441868-1442956)  BMAA1340 (1443264-1444214)  BMAA1341 (1444301-1444927)  BMAA1342 (1445060-1445644)  BMAA1343 (1445706-1446911) | Putative outer membrane porin OpcP  Transcriptional regulator, IclR family  Oxidoreductase, 2OG-Fe(II) oxygenase family  Hypothetical protein  Ser/Thr protein phosphatase family protein |
| 63 | 1533234-1535455 | 1533234-1535455 | 1 | BMAA1425 (1532250-1533785)  BMAA1426 (1533981-1534418)  BMAA1427 (1534415-1534774)  BMAA1428 (1534747-1536465) | Putative methylenomycin A resistance protein  DoxD-like family protein  Hypothetical protein  Lactate permease family protein |
| 64 | 1620999-1623923 | 1620999-1623923 | 1 | BMAA1488 (1620641-1621096)  BMAA1489 (1621232-1621414)  BMAA1490 (1621517-1622719)  BMAA1491 (1622884-1623741) | Hypothetical protein  Hypothetical protein  Acetyl-CoA acetyltransferase  Transcriptional regulator, IclR family |
| 65 | 1654090-1656921 | 1654090-1656921 | 1 | BMAA1524 (1653597-1654160)  BMAA1525 (1654157-1654438)  BMAA1526 (1654622-1655134) | BapC protein  BapB protein  BapA protein |
| 66 | 1787037-1789957 | 1787037-1789957 | 1 | BMAA1643 (1780637-1790623) | Putative peptide synthetase |
| 67 | 1819051-1820905 | 1819051-1820905 | 1 | BMAA1667 (1818099-1819097)  BMAA1668 (1819718-1820287)  BMAA1669 (1820355-1822709) | Patatin-like phospholipase  Putative transporter  Cytochrome P450 |
| 68 | 2064396-2065784 | 2064396-2065784 | 1 | BMAA1884 (2064169-2065173)  BMAA1885 (2065360-2066715) | Urea amidolyase-related protein  Hypothetical protein |
| 69 | 2086339-2089683 | 2086339-2089683 | 1 | BMAA1904 (2085733-2087622)  BMAA1905 (2087653-2088234)  BMAA1906 (2088221-2089186)  BMAA1907 (2089183-2089929) | Hypothetical protein  Hypothetical protein  Hypothetical protein  Hypothetical protein |
| 70 | 2174177-2178481 | 2174177-2178481 | 1 | BMAA1986 (2174881-2175897)  BMAA1987 (2175993-2176994)  BMAA1988 (2176991-2178745) | Putative ADP-heptose--LPS heptosyltransferase II  Glycosyl transferase, group 2 family protein  Carbamoyltransferase family protein |
| 71 | 2200161-2202489 | 2200161-2202489 | 1 | BMAA2010 (2198792-2200192)  BMAA2011 (2200197-2201648)  BMAA2012 (2201650-2202693) | Dihydrolipoamide dehydrogenase  Dihydrolipamide acetyltransferase  2-oxoisovalerate dehydrogenase, E1 component,  beta subunit |
